# Age is Just a Number: Outcomes of Robotic Hiatal Hernia Repair in Octogenarians

**DOI:** 10.1016/j.atssr.2026.02.008

**Published:** 2026-02-25

**Authors:** Samar Semaan, Kendall Howard, Warren C. Naselsky, Ray Chihara, Edward A. Graviss, Min P. Kim

**Affiliations:** 1 Division of Thoracic Surgery, Department of Surgery, Houston Methodist Hospital, Houston, Texas; 2 Department of Pathology and Genomic Medicine, Houston Methodist Hospital, Houston, Texas

## Abstract

**Background:**

Robot-assisted hiatal hernia repair has become increasingly popular in high-risk populations as a relatively low-risk, minimally invasive surgical option. As life expectancy rises, more octogenarians are being considered for surgery despite age-related comorbidities. However, data on the safety and efficacy of robotic repair in this population are limited. This single-center retrospective study evaluates postoperative outcomes and complications after robotic hiatal hernia repair in octogenarians.

**Methods:**

Patients aged ≥80 years who underwent repair between 2017 and 2024 were propensity score–matched in a 1:3 ratio to those <80 years based on sex, body mass index, hernia type, and surgery type. Multivariable regression analyses were used to assess differences in hospital stay and postoperative outcomes, with significance set at *P* < .05.

**Results:**

A total of 302 patients underwent robotic hiatal hernia repair, including 78 patients aged ≥80 years and 224 aged <80 years. The ≥80 group had a median age of 83 years compared with 69 years (*P* < .001), with no differences in sex, body mass index, hernia, or surgery type. There were no significant differences in total hospital hours (30 vs 31, *P* = .11), postoperative events (5.4% vs 7.7%, *P* = .42), or 30-day readmissions (2.2% vs 5.1%, *P* = 24). No mortality was observed in either group. Age was not a significant factor for postoperative events on multivariable regression analysis.

**Conclusions:**

Surgical outcomes did not differ significantly between patients aged ≥80 and <80 years who underwent robot-assisted hiatal hernia repair. Elective robotic-assisted hiatal hernia repair is safe in carefully selected octogenarians.

**In Short**

▪ Age is not a risk factor for complication after robot-assisted hiatal hernia repair.
▪ Carefully selected octogenarians can have excellent outcomes from elective robot-assisted hiatal hernia repair.

The management of hiatal hernias in the elderly is complex. This population faces unique challenges such as multiple comorbidities and physiologic changes, which increase susceptibility to surgical risks.^1^^,^^2^ Surgical treatment of octogenarians has often been reserved for patients with severe symptoms or hiatal hernia complications like gastric volvulus or perforation, with reluctance to treat asymptomatic individuals.^1^^,^^2^ This “wait-and-watch” approach in the elderly has led to more patients undergoing emergency hiatal hernia repair, thus increasing morbidity and mortality rates.^3^ Early elective surgical repair, particularly using minimally invasive laparoscopic approaches, has been shown to mitigate these risks and improve patient outcomes.^3^ Recent studies have found that laparoscopic hiatal hernia repair in elderly patients (>75 years) demonstrated no increased mortality or postoperative complications, and notable improvements in symptomatology and quality of life.^1^^,^^2^

We aimed to determine whether octogenarians who underwent elective repair using robotic technology had similar surgical and functional outcomes to younger patients at our institution.

## Patients and Methods

This study was approved by the institutional review board of the Houston Methodist Hospital Research Institute (Pro00009372). A single-center retrospective cohort study was performed for all patients aged ≥80 years who underwent elective robot-assisted hiatal hernia repair between 2017 and 2024. Octogenarian patient selection at the time of the procedure was based on well-controlled comorbidities, preoperative cardiac clearance demonstrating moderate or lower operative risk, adequate functional status, and willingness to proceed after counseling. All candidates were evaluated and optimized according to our institution’s Enhanced Recovery After Surgery (ERAS) protocol to ensure safe perioperative management.^4^ Data were collected from the Houston Methodist Hospital Thoracic Surgery Foregut database, which captures demographics, surgery characteristics, postoperative events, length of stay at discharge, and survey data on the total Gastroesophageal Reflux Disease Health-Related Quality of Life score (0-50) before and 5 weeks after surgery (Supplemental Figure 1). Additional surgical characteristics and readmission rates were retrospectively obtained from electronic health records, as well as individual cardiac risk stratification data for patients aged ≥80 years. Matching was done for all octogenarians 1:3 with patients <80 years old based on sex, body mass index, and hernia type using nonreplacement and 0.2 caliper width. The number of hours spent in the hospital after surgery, postoperative events, and readmission rates between the 2 groups were also compared.

Prior to surgery, all patients underwent esophagogastroduodenoscopy as well as esophagogram or computed tomography scan. Patients with type I hiatal hernia underwent additional Bravo testing and manometry. Surgery was performed as previously described for robotic hiatal hernia repair for the majority of patients, with differences in pledget use and suture type for crus closure based on surgeon preference.^5^ None of the patients underwent relaxing incision, mesh, or Collis gastroplasty. All patients were on the hiatal hernia repair ERAS protocol.^4^ Additionally, gastroesophageal reflux disease scores before and after surgery were compared.

Differences between groups were assessed using Fisher’s exact test for categorical variables and the Kruskal-Wallis test for continuous variables. Regression analysis was used to determine whether the primary outcomes of total hospital stay and length of stay differed between patients aged ≥80 and <80 years. Multivariable logistic regression was used to evaluate the association between age group and postoperative complications. Candidate variables for inclusion in the model were selected based on clinical relevance and univariable associations with *P* < .10, including sex, body mass index, hernia type, surgery type, and operating surgeon. A purposeful selection strategy was used to construct the final adjusted model. All analyses were performed using Stata 17.0 (StataCorp LLC). Statistical significance was set at *P* < .05.

## Results

A total of 1171 robotic hiatal hernia repairs were performed during the study period. Of these patients, 78 were aged ≥80 years (range, 80-92 years) and were matched (1:3) with 224 patients aged <80 years, yielding a total of 302 patients. The median age of the entire cohort was 72 years, and most were female (86.1%) and White (91.1%). Most patients had type III hiatal hernia (73.5%), and all underwent robotic hiatal hernia repair with majority Toupet fundoplication. Baseline demographic or operative characteristics were not significantly different (Table 1). All octogenarians underwent cardiac risk stratification, with 54% having low risk, 10% having low-to-moderate risk, and 36% having moderate risk of a cardiac event. None were at high risk of cardiac events. All octogenarians were discharged to their prior living arrangements.

**Table 1:** Patient Characteristics

Characteristics	Total	Age <80 y	Age ≥ 80 y	*P* Value
	N = 302	n = 224	n = 78	
Age, y	72.2 (66.0, 80.3)	69.4 (63.3, 74.5)	83.2 (81.7, 86.0)	<.001
BMI (continuous)	26.9 (24.0, 30.1)	27.0 (24.2, 30.2)	26.5 (23.6, 29.8)	.46
Female	260 (86.1)	195 (87.1)	65 (83.3)	.45
Hernia type				.12
I	38 (12.6)	29 (12.9)	9 (11.5)	
II	1 (0.3)	0 (0.0)	1 (1.3)	
III	222 (73.5)	169 (75.4)	53 (67.9)	
IV	41 (13.6)	26 (11.6)	15 (19.2)	
Surgery type				.036
Nissen	5 (1.7)	3 (1.3)	2 (2.6)	
Toupet	264 (87.4)	191 (85.3)	73 (93.6)	
LINX^a^	33 (10.9)	30 (13.4)	3 (3.8)	
Redo hiatal hernia	53 (17.5)	43 (19.2)	10 (12.8)	.23
Surgeon				.003
Surgeon 1	177 (59.0)	135 (60.3)	42 (55.3)	
Surgeon 2	89 (29.7)	72 (32.1)	17 (22.4)	
Surgeon 3	33 (11.0)	17 (7.6)	16 (21.1)	
Surgeon 4	1 (0.3)	0 (0.0)	1 (1.3)	

Values are presented as median (interquartile range) or n (%).

BMI, body mass index.

a LINX Reflux Management System, Johnson & Johnson.

As expected, the octogenarians had a significantly higher median age than the younger group (83.2 vs 69.2 years, *P* < .01). However, there were no statistically significant differences in sex (*P* = .46), median body mass index (*P* = .46), or hiatal hernia type (*P* = .12). Toupet fundoplication was more commonly performed in octogenarians (93.6% vs 85.3%, *P* = .04). The proportion of patients who underwent revisional versus primary surgery was comparable between the age groups (*P* = .23). More patients aged <80 years were operated on by surgeon 1 (*P* < .01, Table 1).

**Figure 1:**
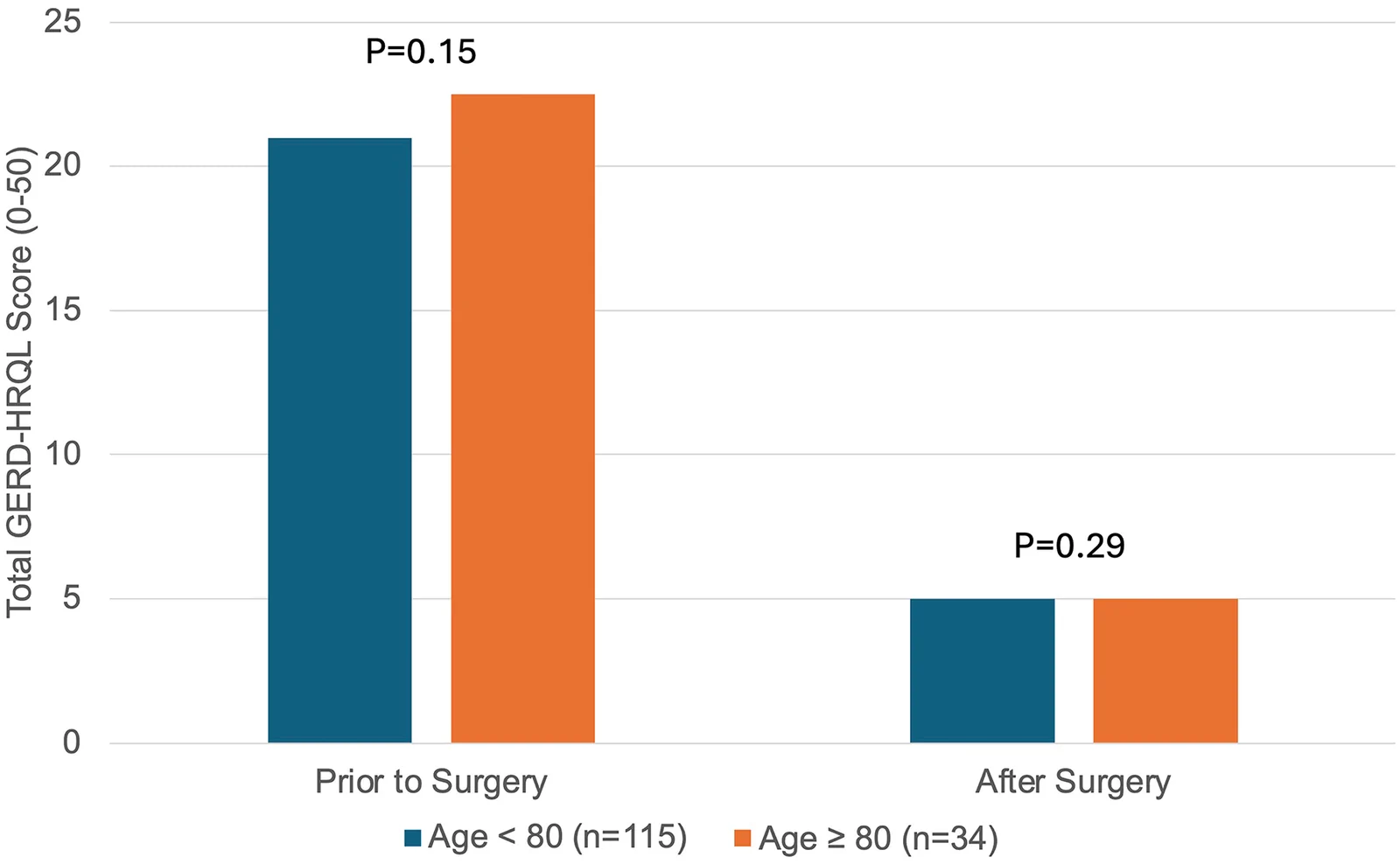
Total Gastroesophageal Reflux Disease Health-Related Quality of Life (GERD-HRQL) score. There was no significant difference between the two age groups prior to (*P* = .15) and after (*P* = .29) surgery.

There was no significant difference between the <80 and ≥80 years old groups in terms of median hours spent in the hospital (30 vs 31, *P* = .11), postoperative events (5.4% vs 7.7%, *P* = .42), and readmission rate (2.2% vs 5.1%, *P* = .24, Table 2). There was no 30-day mortality in either group. Analysis of the median Gastroesophageal Reflux Disease Health-Related Quality of Life score revealed that there was no significant difference between the <80 and ≥80 years groups before (21 vs 22.5, *P* = .15) and after surgery (5 vs 5, *P* = .29, Figure 1). Multivariable analysis of postoperative events showed that age was not associated with complications (odds ratio, 2.01; 95% CI, 0.66, 6.12; *P* = .21). The only factor significantly associated with postoperative events was the procedure performed by surgeon 2 (odds ratio, 4.08; 95% CI 1.31, 12.74; *P* = .02, Table 3).

**Table 2:** Outcomes After Surgery

Characteristics	Total	Age <80	Age ≥ 80	*P* Value
	N = 302	n = 224	n = 78	
Hours in the hospital	30.0 (29.0, 34.0)	30.0 (29.0, 33.0)	31.0 (29.0, 48.0)	.11
Postoperative events	18 (6.0)	12 (5.4)	6 (7.7)	.42
Return to OR	3 (1.0)	3 (1.3)	0 (0.0)	.57
Urinary retention	4 (1.3)	2 (0.9)	2 (2.6)	.28
Atrial fibrillation	2 (0.7)	1 (0.4)	1 (1.3)	.45
Home oxygen	2 (0.7)	2 (0.9)	0 (0.0)	1.00
Diet intolerance	2 (0.7)	1 (0.4)	1 (1.3)	.45
Bronchoscopy	1 (0.3)	1 (0.4)	0 (0.0)	1.00
Myasthenia gravis	1 (0.3)	0 (0.0)	1 (1.3)	.26
Urinary tract infection	1 (0.3)	0 (0.0)	1 (1.3)	.26
Readmission	9 (2.9)	5 (2.2)	4 (5.1)	.24

Values are presented as median (interquartile range) or n (%).

OR, operating room.

**Table 3:** Multivariable Analysis for Postoperative Events

Characteristics	Unadjusted Odds Ratio	Overall *P* Value
	(95% CI)	
Age (<80, ≥80)	2.008 (0.658, 6.123)	.211
Body mass index	0.776 (0.423, 1.424)	.423
Surgeon		
Surgeon 1	Reference	
Surgeon 2	4.081 (1.307, 12.743)	.015
Surgeon 3	0.882 (0.109, 7.133)	.906
Surgeon 4	Empty	

## Comment

Our study suggests that with careful patient selection, robotic hiatal hernia repair can be safely performed in octogenarians. In our cohort, patients aged ≥80 years did not experience higher rates of intraoperative or postoperative complications than their younger counterparts. Age ≥80 years was not an independent predictor of complications. Previous studies on laparoscopic hernia repair have reported similar findings of safety and efficacy in octogenarians, with no increase in intraoperative or postoperative complications or reoperations.^1^^,^^2^ Studies suggest that factors such as frailty are better predictors of adverse outcomes than age itself.^6^ A National Surgical Quality Improvement Program database analysis revealed a stronger correlation between increased complications and composite indices of physiologic frailty than age itself.^6^ Our patients were carefully selected and generally well-optimized, likely contributing to their excellent results. Thus, optimal screening and patient selection have been highlighted as key factors for optimal surgical and postsurgical outcomes.^1^^,^^2^ Our approach prioritized identifying older adults with adequate physiologic reserve and acceptable operative risk, including low-to-moderate cardiac risk on preoperative evaluation and demonstrably good functional status. We also emphasize that careful preoperative assessment and comorbidity management as part of the ERAS program is especially critical for octogenarians to undergo safe hiatal hernia repair.^4^ Together, this helps ensure that candidates are well positioned to benefit from elective repair.

The robotic platform offers technical advantages, such as enhanced 3-dimensional visualization and dexterity, which may be beneficial in more complex dissections. At our institution, robotic hiatal hernia repair had favorable outcomes compared with laparoscopic hiatal hernia repair.^7^ Minimally invasive repair is consistently associated with significantly lower morbidity and fewer complications than open surgery in patients undergoing hiatal hernia repair.^1^^,^^2^ Elective hiatal hernia repair has also been shown to significantly improve outcomes. It allows time for the optimization of cardiopulmonary function and nutritional status, likely reducing perioperative risks. Emergent repair in octogenarians is more common than in younger patients and associated with worse outcomes.^8^ Large database studies demonstrated considerably worse outcomes in octogenarian patients undergoing emergency vs elective repair.^8^^,^^9^ Even after matching, nonelective paraoesophageal hernia repairs had nearly twice the odds of major complications and death compared with elective cases.^9^ This finding is even more pertinent in the elderly, with patients aged ≥70 years having comparable outcomes to younger patients in cases of elective repair but doubled complication and mortality rates in emergency procedures.^10^ Cumulative evidence thus suggests that elective repair with careful assessment can achieve excellent outcomes in the elderly, as frailty rather than age drives risk and well-selected octogenarians have complication rates comparable to younger patients.^1^^,^^6^^,^^10^ This supports the rationale for timely referral rather than prolonged watchful waiting for symptomatic hiatal hernias in this population. The decision must still be individualized; patients with prohibitive comorbidities or limited functional status may still defer surgery.

The major limitation of our study is its retrospective design and single-center setting. However, the inclusion of a large octogenarian cohort treated by multiple surgeons may provide meaningful insight into the management of this population. Other factors such as frailty may have also influenced outcomes but were not captured in this retrospective analysis. Furthermore, all patients underwent robotic hiatal hernia repair, limiting the generalizability of these findings to laparoscopic or open approaches. Future prospective studies should continue to refine the criteria for surgery in the elderly and further compare minimally invasive approaches in this growing patient population.

In conclusion, robotic hiatal hernia repair is safe and effective in carefully selected octogenarians under the ERAS protocol, with age alone not precluding surgery. Given the poor outcomes associated with emergent hiatal hernia repair in octogenarians, patients with low to moderate cardiac risk should be considered for elective robotic repair.
