# Supplementary material for: Age is Just a Number: Outcomes of Robotic Hiatal Hernia Repair in Octogenarians
Source: Ann Thorac Surg Short Rep. 2026 Feb 25;4(3):972–6. doi: 10.1016/j.atssr.2026.02.008 (PMC13555422; doi:10.1016/j.atssr.2026.02.008)
Supplement: Supplementary file 1 [file mmc1.docx]

**Thoracic Surgery Clinic**

*Reflux and hiatal hernia*

Questionnaire after surgery

Scale

0 – No symptoms

1 – Symptoms noticeable, but not bothersome

2 – Symptoms noticeable and bothersome, but not every day

3 – Symptoms bothersome every day

4 – Symptoms affect daily activities

5 – Symptoms are incapacitating, unable to do daily activity

1. How bad is your heartburn?	0	1	2	3	4	5

2. Heartburn when lying down?	0	1	2	3	4	5

3. Heartburn when standing up?	0	1	2	3	4	5

4. Heartburn after meals?	0	1	2	3	4	5

5. Does heartburn change your diet?	0	1	2	3	4	5

6. Does heartburn wake you from sleep?	0	1	2	3	4	5

7. Do you have difficulty swallowing?	0	1	2	3	4	5

8. Do you have pain with swallowing?	0	1	2	3	4	5

9. Do you have bloating or gassy feeling?	0	1	2	3	4	5

10. If you take medication, does this	0	1	2	3	4	5

affect your daily life?

11. How satisfied are you with your present	Satisfied	Neutral	Dissatisfied

condition?

12. If you could choose it today, would you do surgery again?	YES	NO

13. If you could classify your symptom improvement between 0 to 10 what grade would you give?

0	1	2	3	4	5	6	7	8	9	10
